# Spirometry documentation gaps and reporting delays in a COPD reporting system: implications for surveillance data quality

**DOI:** 10.3389/fmed.2026.1955292

**Published:** 2026-10-14

**Authors:** Changfu Chen, Jiali Zhou, Hongying Zhang

**Affiliations:** 1 Department of Science and Education, Rehabilitation Hospital of Chongqing Medical University, Chongqing, China; 2 School of Nursing, Chongqing Medical University, Chongqing, China

**Keywords:** chronic obstructive pulmonary disease, hospital reporting system, public health surveillance, registry data quality, reporting delay, spirometry

## Abstract

**Background:**

Chronic obstructive pulmonary disease (COPD) reporting systems can support public health surveillance and quality improvement, but their value depends on complete diagnostic documentation and timely data entry.

**Methods:**

We conducted a retrospective cross-sectional analysis of records from the COPD case-reporting module of the Chongqing municipal chronic disease monitoring platform, provided by the Chongqing Center for Disease Control and Prevention, with clinical visit dates in 2024. Records from persons younger than 40 years were excluded. Acute exacerbation of COPD (AECOPD) was classified from explicit diagnostic text indicating acute exacerbation. Spirometry documentation was defined as diagnostic-basis documentation of pulmonary function testing or a recorded pre- or post-bronchodilator FEV_1_/FVC value. Reporting delay was calculated from the clinical visit date to the system-recorded entry timestamp; >7 days was prespecified as an operational quality threshold rather than a statutory reporting deadline, with >14- and >30-day thresholds examined in sensitivity analyses.

**Results:**

Among 1,076 included records, median age was 73.2 years (IQR 68.1–80.3), and 829 (77.0%) records were for males. AECOPD was explicitly recorded in 357 (33.2%) records. Any spirometry documentation was present in 195 (18.1%) records, diagnostic-basis spirometry in 167 (15.5%), and post-bronchodilator FEV_1_/FVC in 133 (12.4%). Older age was associated with higher odds of absent spirometry documentation (OR 1.03 per year, 95% CI 1.01–1.04), whereas municipal-hospital records had lower odds than county/district-hospital records (OR 0.52, 95% CI 0.33–0.82). Median reporting delay was 19 days (IQR 9–32.5), and 851 (79.1%) records were entered >7 days after the visit.

**Conclusion:**

Gaps in spirometry documentation and delays in reporting-system entry were common. These findings identify actionable data-quality targets for COPD surveillance systems, particularly structured spirometry-status fields, explicit reasons for unavailable testing, and routine timeliness monitoring. Because the study evaluated submitted records from a single municipal reporting system, the findings should not be interpreted as estimates of clinical spirometry utilization or population-level case capture and should be validated in other reporting settings.

## Introduction

Chronic obstructive pulmonary disease (COPD) is a common, preventable, and treatable respiratory condition associated with substantial mortality, disability, and health-system burden (1). Global burden analyses continue to identify COPD as a leading cause of death and disability (2, 3). China bears a large share of this burden: national population-based studies have documented a high prevalence of spirometry-defined COPD among adults aged 40 years or older (4, 5), and recent analyses show substantial continuing morbidity, mortality, and regional inequality (6).

Improving early detection and standardized management has become an explicit priority in China, including national screening and tiered-care initiatives (7). Important gaps remain across hospital levels. In one Chinese tiered-care study, pulmonary function testing at first COPD diagnosis was reported in 99.0% of patients in teaching hospitals, 69.4% in second-tier hospitals, and 29.9% in community hospitals; testing within the previous year was reported in 67.3%, 24.9%, and 28.9%, respectively (8). The same study found that the proportion of patients who either did not use inhaled drugs or used them irregularly was 2.0%, 24.6%, and 78.8% across these hospital tiers (8). A large prospective real-world study also documented incomplete guideline-concordant maintenance treatment and mean adherence to long-acting inhalers of 59.0%, indicating persistent treatment and follow-up challenges (9).

Spirometry is central to COPD diagnosis. The Global Initiative for Chronic Obstructive Lung Disease (GOLD) strategy requires demonstration of persistent airflow limitation, usually a post-bronchodilator forced expiratory volume in 1 s/forced vital capacity (FEV_1_/FVC) ratio <0.70 (10). The American Thoracic Society/European Respiratory Society (ATS/ERS) technical statement defines quality standards for acceptable and repeatable spirometry (11), while recent GOLD discussions emphasize complementary roles for pre- and post-bronchodilator testing in clinical workflows (12). Despite these recommendations, confirmatory spirometry is frequently absent from routine-care records (13–15). Systematic reviews further show that both undiagnosed airflow obstruction and COPD labeling without spirometric confirmation occur in practice, emphasizing the value of objective diagnostic documentation (16, 17).

Acute exacerbation of COPD (AECOPD) is clinically important because exacerbations are associated with hospitalization, healthcare resource use, and adverse outcomes (18–20). Clinical registries and disease reporting systems can support quality assessment, benchmarking, and real-world research when key variables are captured completely and promptly (21–23). Completeness, accuracy, consistency, and timeliness are recognized dimensions of health-data quality (24–26), and timeliness is specifically relevant to public-health surveillance (27).

Evidence that simultaneously evaluates COPD diagnostic-documentation completeness and reporting timeliness across hospital tiers in real-world surveillance systems remains limited. We therefore used municipal COPD reporting-system data to quantify spirometry and other diagnostic documentation, examine factors associated with recorded AECOPD classification, and evaluate reporting-system entry delay and its associated factors. The study was designed to identify measurable and potentially modifiable data-quality indicators that may inform COPD surveillance and registry improvement, rather than to estimate clinical spirometry utilization, diagnostic accuracy, or population-level completeness of COPD case capture. The analysis was conducted using the Chongqing municipal COPD reporting system, which was formally implemented from 2017 and had expanded across the municipality by 2019 (28). More recent official reports describe continued platform optimization and medical-public-health data integration intended to improve reporting quality (29, 30).

## Materials and methods

### Study design and setting

This retrospective cross-sectional study used records from the COPD case-reporting module of the Chongqing municipal chronic disease monitoring platform. The analytical dataset was provided by the Chongqing Center for Disease Control and Prevention (Chongqing CDC) for research use and included records with clinical visit dates in 2024. Data collection and transfer of the de-identified analytical dataset for this study took place between April and May 2026. COPD case registration and reporting in Chongqing has been implemented since 2017, and the municipal monitoring platform forms part of the city's chronic disease surveillance and management system (28–30). The study was reported in accordance with the STROBE recommendations for cross-sectional studies (31, 32); the completed STROBE checklist is provided as Supplementary Appendix S1.

### Data source and participants

The study team received a de-identified analytical extract from Chongqing CDC. The extract contained demographic fields, occupation-related fields, diagnosis text, diagnostic basis, spirometry fields, hospital level, clinical visit date, a system-recorded entry timestamp, and mortality-related fields where available. The dataset therefore represents COPD records submitted to the municipal monitoring platform and included in the research extract. Metadata describing the total number of otherwise eligible COPD encounters that were never submitted, the number of individual contributing hospitals, and complete case-capture coverage were not available. Accordingly, this study evaluates completeness and timeliness within submitted reporting-system records and cannot estimate the completeness of COPD case reporting across all eligible healthcare encounters.

Records were screened for duplicate reporting-system identifiers and for age eligibility. Records from persons aged <40 years were excluded to align with adult COPD epidemiologic conventions and to reduce inclusion of atypical or miscoded cases. The analytical extract did not include a separate persistent patient identifier; therefore, the absence of duplicate reporting-system identifiers could not establish that all records represented unique individuals. Record-selection counts are reported in the Results and Figure 1.

**Figure 1:**
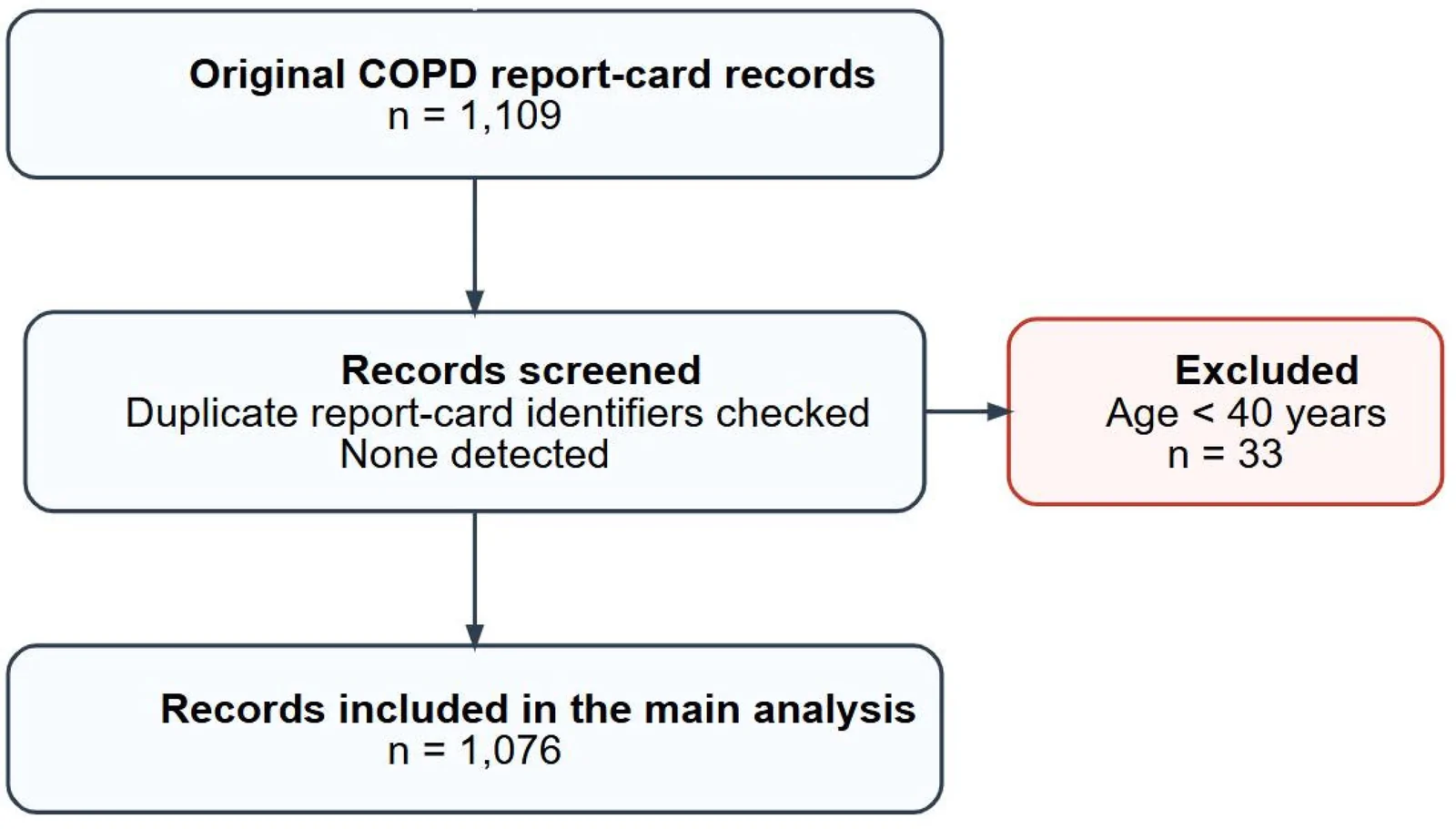
Record-selection flowchart. The original hospital COPD reporting-system dataset contained 1,109 records. Records were checked for duplicate reporting-system identifiers and screened for age eligibility. After excluding 33 records for persons younger than 40 years, 1,076 records were included in the main analysis.

### Variable definitions

Age was calculated from birth date and visit date where needed and grouped as <50, 50–59, 60–69, 70–79, and >=80 years. Sex, ethnicity, marital status, occupation, and hospital level were harmonized from the original reporting-system fields. Occupation was recoded using both the industry and specific-occupation fields into agriculture/forestry/fishery, unemployed, other occupations, or other/uncategorized; the recoding scheme and distribution are shown in Supplementary Table S4.

AECOPD was classified when the original diagnostic text explicitly indicated chronic obstructive pulmonary disease with acute exacerbation. Records containing COPD with acute lower respiratory infection but without explicit acute-exacerbation wording were classified as non-AECOPD in the primary analysis and included as AECOPD only in a broader sensitivity definition. The complete diagnostic-text dictionary and classification rules are provided in Supplementary Table S2. Diagnostic-basis text was parsed to identify clinical symptoms, spirometry/pulmonary function testing, chest x-ray, chest computed tomography (CT), and laboratory tests.

Any spirometry documentation was defined as at least one of the following: diagnostic-basis text indicating pulmonary function testing, a recorded pre-bronchodilator FEV_1_/FVC value, or a recorded post-bronchodilator FEV_1_/FVC value. Sentinel values used to denote unavailable spirometry measurements were treated as missing numeric measurements rather than physiological values; data-quality checks are summarized in Supplementary Table S1. This definition measures reporting-system documentation and does not establish whether spirometry was or was not performed clinically (10, 33).

Post-bronchodilator airflow obstruction was described among records with valid post-bronchodilator FEV_1_/FVC measurements using the fixed-ratio threshold <0.70 (Supplementary Table S5). A descriptive sensitivity analysis was also performed among records with documented post-bronchodilator FEV_1_/FVC <0.70 (Supplementary Table S12). The reporting system did not permit determination of whether records with FEV_1_/FVC >=0.70 had prior confirmatory spirometry, testing during clinical stability, or other evidence supporting the registered COPD diagnosis.

Reporting delay was defined as the number of calendar days between the clinical visit date and the system-recorded entry timestamp in the analytical extract. Publicly available Chongqing documents confirm municipal COPD case reporting and emphasize surveillance quality and information-platform development (28–30); however, we did not identify a publicly available municipal regulation establishing a universal 7-day deadline for COPD record entry. Therefore, >7 days was prespecified as an operational quality threshold for the primary analysis rather than interpreted as noncompliance with a statutory or municipal reporting deadline. Alternative thresholds of >14 and >30 days were examined in sensitivity models (Supplementary Table S7). Because the exported timestamp did not identify whether it represented initial record creation, formal submission, administrative approval, or subsequent modification, the outcome is conservatively termed reporting-system entry delay rather than a specific workflow interval.

### Missing data and data quality

Variables were examined for missingness, sentinel values, and logical inconsistencies before analysis (Supplementary Table S1). The analytical dataset was de-identified before statistical analysis; direct personal identifiers were not used in the analytical models, and reporting-system identifiers were used only for duplicate-record checks. No record-level identifiers are presented in the manuscript or supplementary materials. Missing spirometry documentation was analyzed as a reporting-system data-quality outcome and was not imputed.

Dates with implausible ordering were flagged. Because first-diagnosis dates included a small number of implausible or inconsistent values, diagnostic delay from first diagnosis to the current visit was not used as a primary endpoint (34). Reporting-delay distributions, extreme-delay frequencies, and temporal patterns were examined separately (Supplementary Table S9; Supplementary Figures S1–S3).

### Statistical analysis

Continuous variables are summarized as medians and interquartile ranges because process-time variables were skewed. Categorical variables are summarized as counts and percentages. Group comparisons between records classified as AECOPD and non-AECOPD used the Wilcoxon rank-sum test for continuous variables and Pearson's chi-square test for categorical variables.

Three multivariable logistic regression models were fitted for recorded AECOPD classification, absent spirometry documentation, and reporting delay >7 days. Covariates were selected *a priori* on clinical and registry relevance and were entered simultaneously; no stepwise variable-selection procedure was used. Model sample sizes and event counts, assessment of age linearity in the logit, multicollinearity checks, and influence diagnostics are summarized in Supplementary Table S13. Individual hospital identifiers were unavailable, so within-hospital clustering could not be modeled; hospital-level coefficients therefore represent associations with hospital tier, not independent institutional effects.

Sensitivity analyses included a broader AECOPD definition (Supplementary Table S6), alternative reporting-delay thresholds (Supplementary Table S7), adjusted predicted probabilities and absolute probability differences for the common >7-day delay outcome (Supplementary Table S8; Supplementary Figure S4), exclusion of extreme delays (Supplementary Table S10), alternative handling of occupation (Supplementary Table S11), and a natural-cubic-spline analysis of age for absent spirometry documentation (Supplementary Table S13; Supplementary Figure S5). Statistical analyses were conducted using R version 4.5.2. Results are presented as odds ratios (ORs) with 95% confidence intervals (CIs), and two-sided *P* values are reported.

## Results

### Patient selection and baseline characteristics

Among 1,109 original reporting-system records, 33 were excluded because age was <40 years, leaving 1,076 records for analysis (Figure 1). No duplicate reporting-system identifiers were identified. Median age was 73.2 years (IQR 68.1–80.3), and 829 (77.0%) records were for males. Most records indicated Han ethnicity (1,056, 98.1%). AECOPD was explicitly recorded in 357 (33.2%) records.

AECOPD classification differed by marital status, occupation group, hospital level, clinical-symptom documentation, and chest CT documentation (Table 1). The proportion classified as AECOPD was higher in county/district and township hospitals than in municipal and provincial hospitals. The proportion increased numerically across age groups, from 22.2% among records for persons aged <50 years to 35.9% among those aged >=80 years (Figure 2). Diagnostic-text frequencies and the broader AECOPD sensitivity definition are shown in Supplementary Tables S2, S6.

**Table 1:** Characteristics of reporting-system records by AECOPD classification.

Variable	Overall	Non-AECOPD	AECOPD	*P* value
Age, years	73.2 (68.1, 80.3)	72.9 (68.0, 79.8)	74.1 (68.5, 81.2)	0.14
Age group, years				0.6
<50	9 (0.8%)	7 (1.0%)	2 (0.6%)	
50–59	26 (2.4%)	19 (2.6%)	7 (2.0%)	
60–69	295 (27%)	204 (28%)	91 (25%)	
70–79	434 (40%)	289 (40%)	145 (41%)	
≥80	312 (29%)	200 (28%)	112 (31%)	
Sex				0.4
Male	829 (77%)	555 (77%)	274 (77%)	
Female	246 (23%)	164 (23%)	82 (23%)	
Unknown	1 (<0.1%)	0 (0%)	1 (0.3%)	
Ethnicity				0.3
Han	1,056 (98%)	703 (98%)	353 (99%)	
Other ethnicities	20 (1.9%)	16 (2.2%)	4 (1.1%)	
Marital status				0.016
Divorced	9 (0.8%)	7 (1.0%)	2 (0.6%)	
Married	715 (66%)	497 (69%)	218 (61%)	
Unknown	282 (26%)	166 (23%)	116 (32%)	
Unmarried	19 (1.8%)	15 (2.1%)	4 (1.1%)	
Widowed	51 (4.7%)	34 (4.7%)	17 (4.8%)	
Occupation group				0.013
Agriculture/forestry/fishery	63 (5.9%)	31 (4.3%)	32 (9.0%)	
Other occupations	93 (8.6%)	62 (8.6%)	31 (8.7%)	
Other/uncategorized	770 (72%)	530 (74%)	240 (67%)	
Unemployed	150 (14%)	96 (13%)	54 (15%)	
Hospital level				<0.001
County/district hospital	678 (63%)	417 (58%)	261 (73%)	
Municipal hospital	152 (14%)	119 (17%)	33 (9.2%)	
Other	4 (0.4%)	2 (0.3%)	2 (0.6%)	
Provincial hospital	191 (18%)	154 (21%)	37 (10%)	
Township hospital	51 (4.7%)	27 (3.8%)	24 (6.7%)	
Any spirometry documentation	195 (18%)	131 (18%)	64 (18%)	>0.9
Pre-bronchodilator FEV_1_/FVC, %	58.8 (54.0, 64.7)	57.7 (49.8, 64.7)	60.1 (56.2, 63.1)	0.2
Unknown	933	627	306	
Post-bronchodilator FEV_1_/FVC, %	63.9 (53.1, 67.0)	63.4 (49.5, 67.0)	64.0 (60.0, 66.3)	0.2
Unknown	943	636	307	
Clinical symptoms recorded	904 (84%)	574 (80%)	330 (92%)	<0.001
Spirometry recorded as diagnostic basis	167 (16%)	118 (16%)	49 (14%)	0.3
Chest x-ray recorded	198 (18%)	123 (17%)	75 (21%)	0.14
Chest CT recorded	446 (41%)	278 (39%)	168 (47%)	0.010
Laboratory tests recorded	175 (16%)	112 (16%)	63 (18%)	0.4
Reporting delay, days	19.0 (9.0, 32.5)	18.0 (9.0, 31.0)	20.0 (10.0, 37.0)	0.2
Reporting delay category				0.4
≤7 days	225 (21%)	153 (21%)	72 (20%)	
8–14 days	193 (18%)	134 (19%)	59 (17%)	
15–30 days	369 (34%)	251 (35%)	118 (33%)	
>30 days	289 (27%)	181 (25%)	108 (30%)	
Invalid/missing	0 (0%)	0 (0%)	0 (0%)	
Death recorded	26 (2.5%)	18 (2.5%)	8 (2.3%)	>0.9
Unknown	18	11	7	

Values are median (Q1, Q3) or *n* (%). *P* values are from Wilcoxon rank-sum tests for continuous variables and Pearson chi-square tests for categorical variables. AECOPD, acute exacerbation of chronic obstructive pulmonary disease; CT, computed tomography; FEV_1_/FVC, forced expiratory volume in 1 s/forced vital capacity.

**Figure 2:**
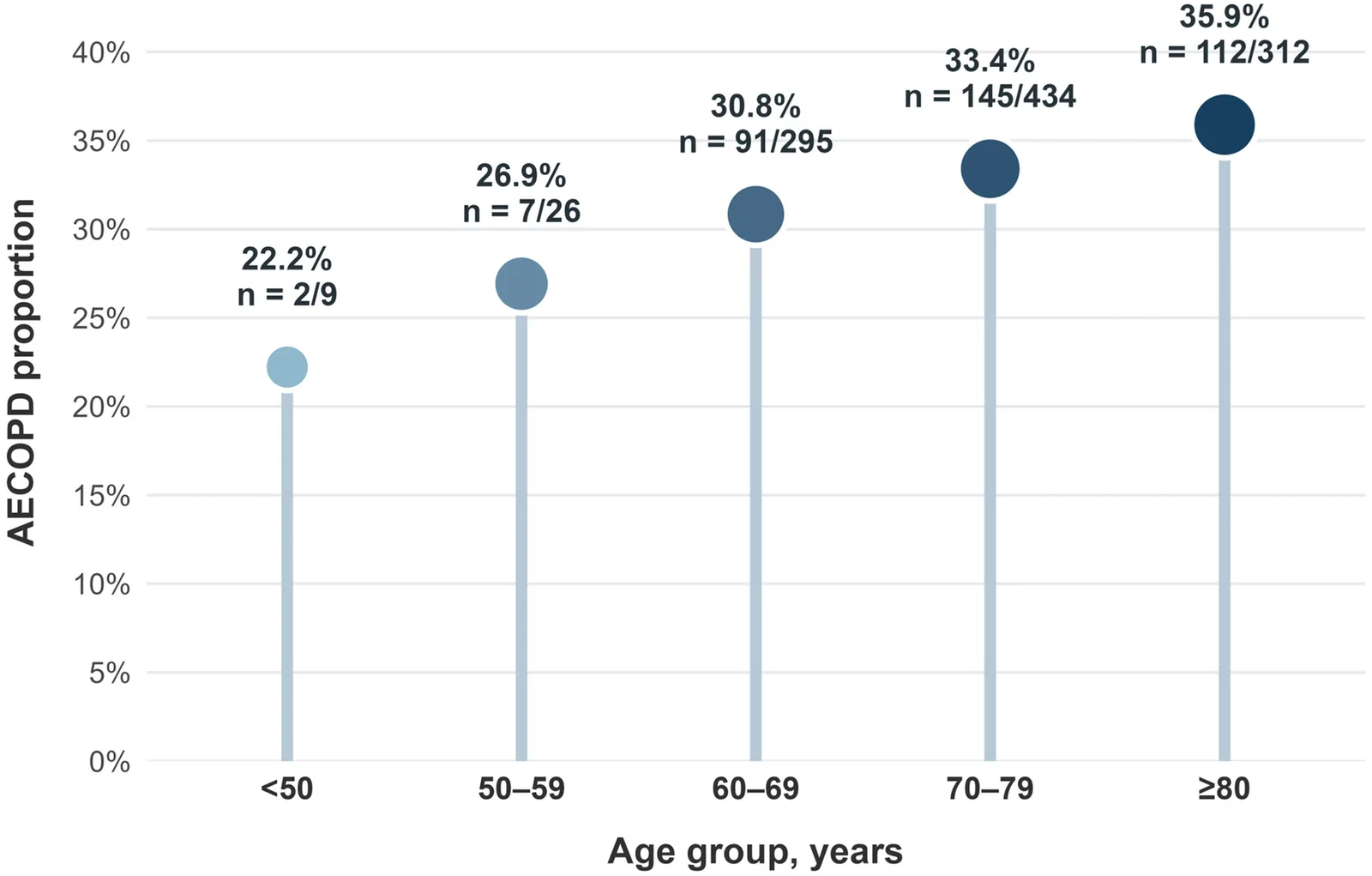
Recorded AECOPD classification by age group. Lollipop plot showing the proportion of reporting-system records classified as acute exacerbation of chronic obstructive pulmonary disease (AECOPD) within each age group. Points indicate the observed proportions, vertical stems extend from zero to each estimate, and labels show the percentage and the number of AECOPD records relative to the total number of records in each age group.

### Diagnostic documentation

Diagnostic documentation relied primarily on clinical symptoms, recorded in 904 (84.0%) records, followed by chest CT in 446 (41.4%), chest x-ray in 198 (18.4%), laboratory tests in 175 (16.3%), and spirometry recorded as a diagnostic basis in 167 (15.5%). Any spirometry documentation was present in 195 (18.1%) records, whereas a valid post-bronchodilator FEV_1_/FVC measurement was recorded in 133 (12.4%). Among these 133 records, 114 (85.7%) had post-bronchodilator FEV_1_/FVC <0.70 and 19 (14.3%) had values >=0.70 (Supplementary Table S5). Data-quality checks are summarized in Supplementary Table S1.

Any spirometry documentation varied by hospital level: 17.6% in county/district hospitals, 23.7% in municipal hospitals, 15.2% in provincial hospitals, and 19.6% in township hospitals. Chest CT documentation was more common in municipal and provincial hospitals, whereas chest x-ray documentation was more common in provincial and township hospitals (Table 2; Figure 3; Supplementary Table S3). The descriptive analysis restricted to records with documented post-bronchodilator FEV_1_/FVC <0.70 included 113 model-eligible records; 43 (38.1%) were classified as AECOPD and 83 (73.5%) had reporting delay >7 days (Supplementary Table S12).

**Table 2:** Diagnostic documentation by hospital level.

Diagnostic item	County/district hospital	Municipal hospital	Other	Provincial hospital	Township hospital
Any spirometry documentation	119/678 (17.6%)	36/152 (23.7%)	1/4 (25.0%)	29/191 (15.2%)	10/51 (19.6%)
Chest CT recorded	256/678 (37.8%)	79/152 (52.0%)	0/4 (0.0%)	103/191 (53.9%)	8/51 (15.7%)
Chest x-ray recorded	101/678 (14.9%)	8/152 (5.3%)	0/4 (0.0%)	69/191 (36.1%)	20/51 (39.2%)
Clinical symptoms recorded	618/678 (91.2%)	104/152 (68.4%)	3/4 (75.0%)	138/191 (72.3%)	41/51 (80.4%)
Laboratory tests recorded	119/678 (17.6%)	18/152 (11.8%)	0/4 (0.0%)	28/191 (14.7%)	10/51 (19.6%)
Post-bronchodilator FEV_1_/FVC recorded	76/678 (11.2%)	31/152 (20.4%)	1/4 (25.0%)	18/191 (9.4%)	7/51 (13.7%)
Pre-bronchodilator FEV_1_/FVC recorded	80/678 (11.8%)	30/152 (19.7%)	1/4 (25.0%)	25/191 (13.1%)	7/51 (13.7%)
Spirometry recorded as diagnostic basis	97/678 (14.3%)	31/152 (20.4%)	1/4 (25.0%)	28/191 (14.7%)	10/51 (19.6%)

Values are n/N (%). CT, computed tomography; FEV_1_/FVC, forced expiratory volume in 1 s/forced vital capacity.

**Figure 3:**
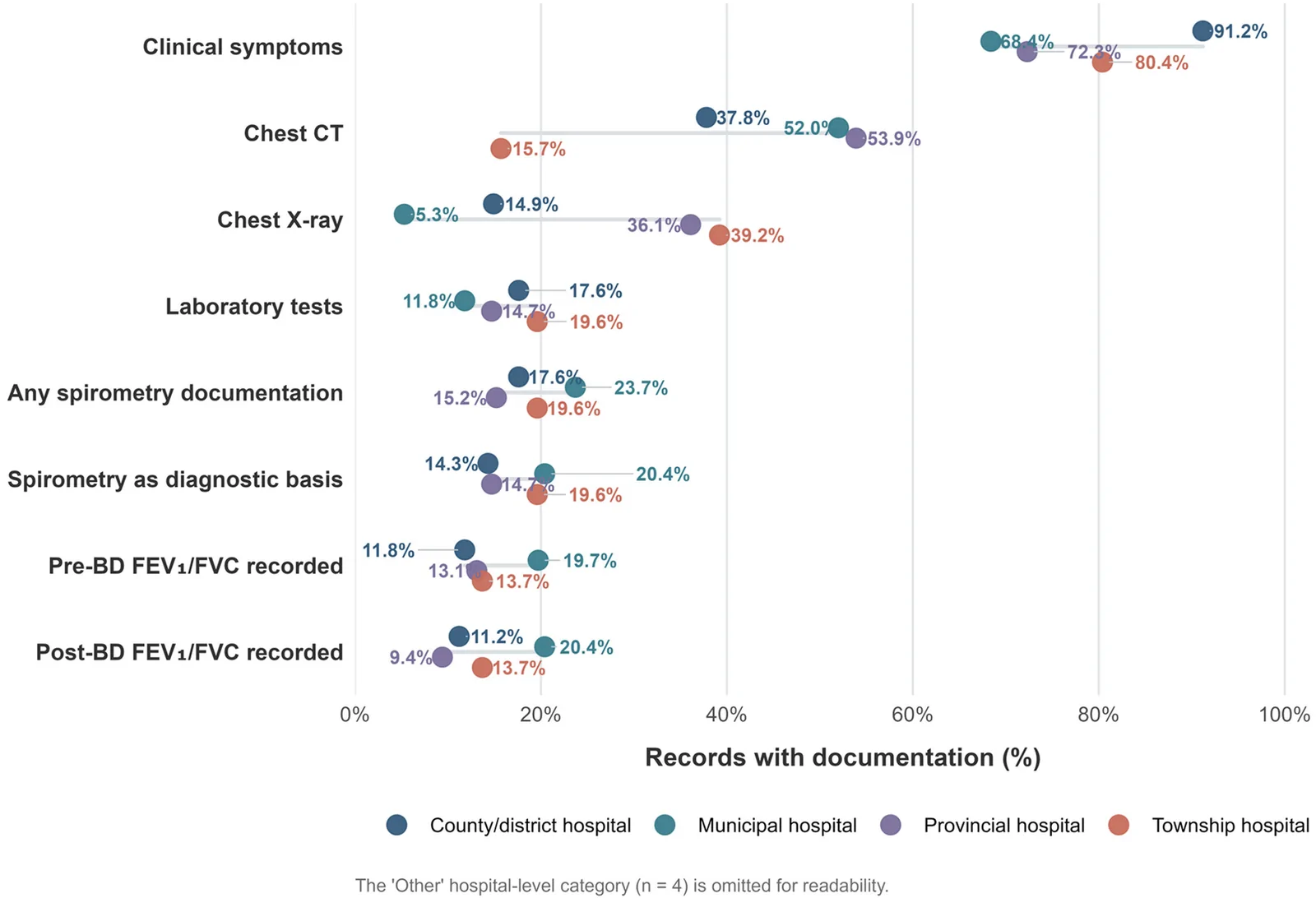
Diagnostic documentation by hospital level. Cleveland dot plot comparing the percentage of reporting-system records with each diagnostic-documentation item across county/district, municipal, provincial, and township hospitals. Colored points represent hospital levels and labels show the corresponding percentages. The sparse Other hospital-level category (*n* = 4) was omitted from the figure for readability but was retained in Table 2.

### Reporting delay

Median reporting delay was 19 days (IQR 9–32.5; Figure 4; Supplementary Figure S1). Overall, 851 (79.1%) records were entered >7 days after the visit, 658 (61.2%) > 14 days, and 289 (26.9%) > 30 days. The 99th percentile was 264 days; 123 records (11.4%) had delays >90 days and 79 (7.3%) had delays >180 days (Supplementary Table S9). Long delays were temporally concentrated, including 160 records entered in September 2024 with a median delay of 162.5 days, and were disproportionately observed in county/district hospitals (Supplementary Figures S2, S3). These patterns are compatible with retrospective or batch entry, although workflow metadata were unavailable to confirm the mechanism.

**Figure 4:**
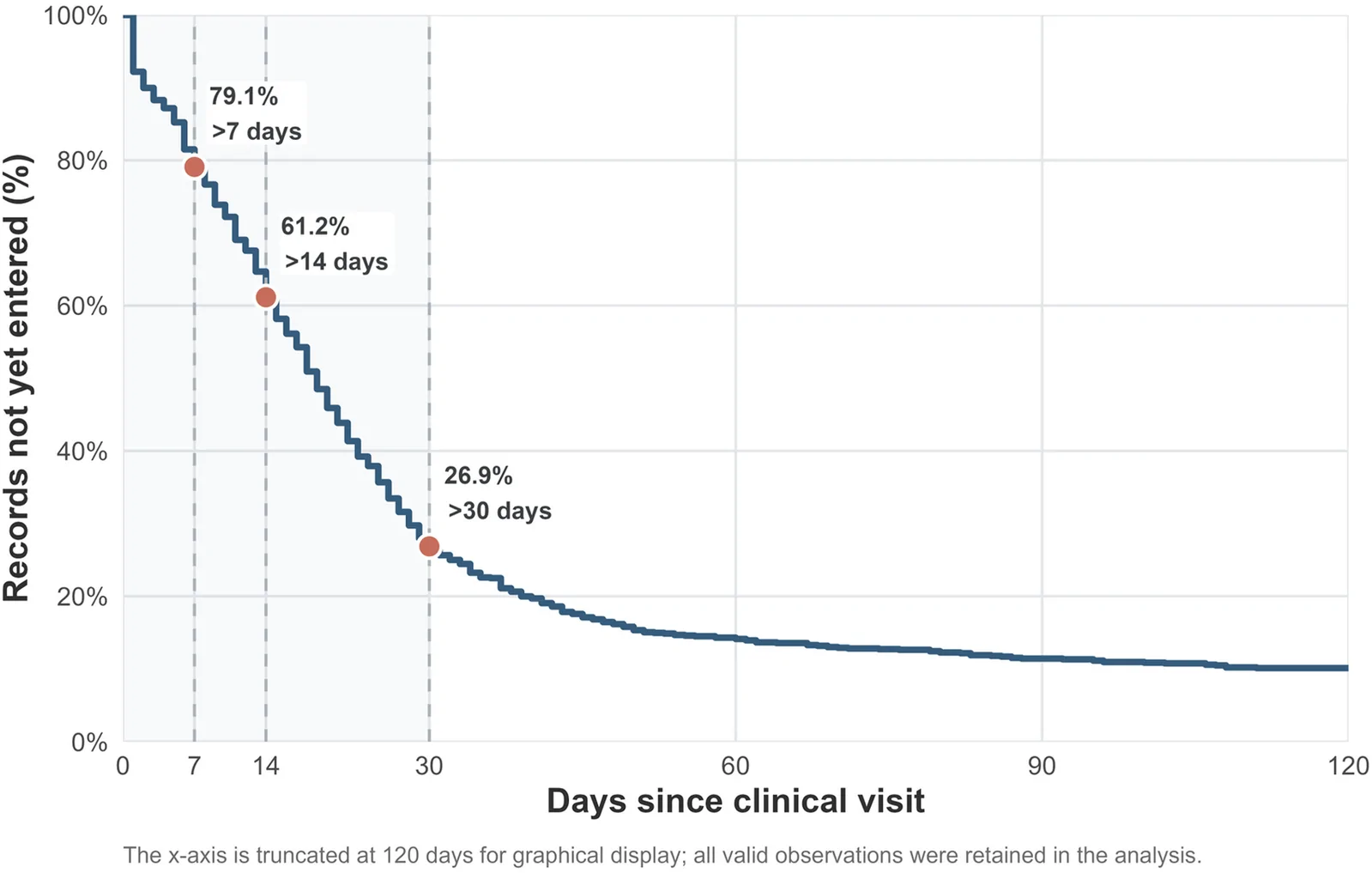
Reporting-system entry delay. Reverse empirical cumulative distribution curve showing the proportion of records remaining unentered in the reporting system as a function of days since the clinical visit. At 7, 14, and 30 days, 79.1%, 61.2%, and 26.9% of records, respectively, remained unentered. Vertical dashed lines indicate these prespecified thresholds. The *x*-axis was truncated at 120 days for graphical display only; all valid observations were retained in the analysis.

### Multivariable regression analyses

In the adjusted AECOPD model, municipal-hospital records (OR 0.42, 95% CI 0.27–0.65) and provincial-hospital records (OR 0.32, 95% CI 0.21–0.47) had lower odds of recorded AECOPD classification than county/district-hospital records. Chest CT documentation was associated with higher odds of recorded AECOPD classification (OR 1.69, 95% CI 1.28–2.25) (Table 3; Figure 5A). Under the broader AECOPD sensitivity definition, the municipal-hospital association remained in the same direction, whereas the provincial-hospital estimate was attenuated (Supplementary Table S6).

**Table 3:** Multivariable logistic regression models.

Variable	AECOPD OR (95% CI); P	No spirometry documentation OR (95% CI); P	Reporting delay >7 days OR (95% CI); P
Age, per year	1.01 (0.99–1.02); 0.312	1.03 (1.01–1.04); 0.002	1.00 (0.98–1.01); 0.749
Female vs. male	1.06 (0.77–1.44); 0.726	0.79 (0.55–1.15); 0.216	0.81 (0.57–1.15); 0.228
Agriculture/forestry/fishery vs. other/uncategorized	1.54 (0.83–2.85); 0.169	0.47 (0.23–1.01); 0.045	1.31 (0.64–2.89); 0.476
Unemployed vs. other/uncategorized	1.08 (0.71–1.62); 0.718	0.26 (0.17–0.40); <0.001	1.16 (0.73–1.89); 0.529
Other occupations vs. other/uncategorized	0.87 (0.53–1.40); 0.579	0.28 (0.17–0.46); <0.001	0.98 (0.58–1.72); 0.938
Township hospital vs. county/district hospital	1.22 (0.62–2.36); 0.562	1.51 (0.69–3.56); 0.320	0.69 (0.32–1.56); 0.355
Municipal hospital vs. county/district hospital	0.42 (0.27–0.65); <0.001	0.52 (0.33–0.82); 0.005	0.57 (0.38–0.86); 0.007
Provincial hospital vs. county/district hospital	0.32 (0.21–0.47); <0.001	1.30 (0.81–2.14); 0.296	2.45 (1.51–4.13); <0.001
Spirometry documented vs. not documented	0.93 (0.65–1.33); 0.702	—	0.88 (0.60–1.32); 0.529
Chest CT recorded vs. not recorded	1.69 (1.28–2.25); <0.001	1.19 (0.84–1.69); 0.321	0.54 (0.40–0.75); <0.001
Chest x-ray recorded vs. not recorded	1.36 (0.95–1.93); 0.091	0.74 (0.49–1.15); 0.172	0.59 (0.40–0.88); 0.010
Reporting delay >7 days vs. ≤7 days	1.20 (0.86–1.68); 0.278	—	—
AECOPD vs. non-AECOPD	—	1.06 (0.75–1.52); 0.739	1.20 (0.86–1.68); 0.282

Models included 1,071 records after excluding one record with unknown sex and four records in the sparse Other hospital-level category. All prespecified covariates were entered simultaneously. Reference groups were male sex, other/uncategorized occupation, county/district hospital, and absence of the relevant documentation indicator. AECOPD, acute exacerbation of chronic obstructive pulmonary disease; CI, confidence interval; CT, computed tomography; OR, odds ratio.

**Figure 5:**
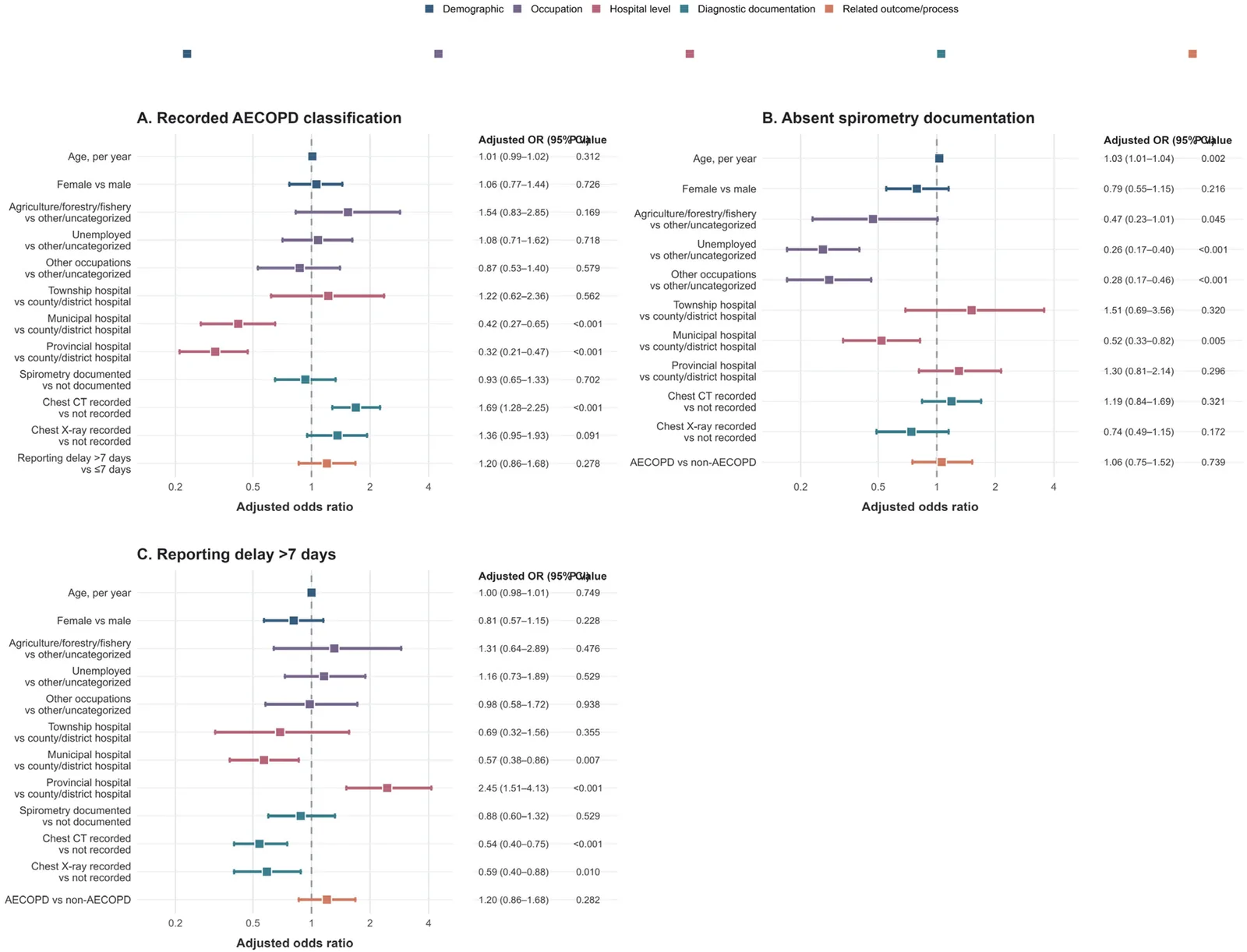
Multivariable factors associated with COPD reporting-system outcomes. Forest plots show adjusted odds ratios (ORs), 95% confidence intervals (CIs), and *P* values from the three prespecified multivariable logistic regression models: **(A)** recorded acute exacerbation of chronic obstructive pulmonary disease (AECOPD) classification; **(B)** absent spirometry documentation; and **(C)** reporting-system entry delay >7 days. Squares indicate adjusted ORs, horizontal lines indicate 95% CIs, and the dashed vertical line indicates an OR of 1. Colors distinguish demographic, occupation, hospital-level, diagnostic-documentation, and timeliness covariates. Reference categories are shown in the corresponding variable labels.

In the model for absent spirometry documentation, older age was associated with higher odds (OR 1.03 per year, 95% CI 1.01–1.04), and municipal-hospital records had lower odds than county/district-hospital records (OR 0.52, 95% CI 0.33–0.82) (Table 3; Figure 5B). Occupation-related coefficients were not interpreted as socioeconomic effects because the other/uncategorized reference category remained heterogeneous; results were materially similar in sensitivity analyses that changed occupation handling (Supplementary Tables S4, S11). A spline sensitivity analysis suggested modest nonlinearity in the age association (likelihood-ratio test *P* = 0.031), with marginally standardized predicted probabilities generally increasing with age and wider uncertainty at the extremes (Supplementary Table S13; Supplementary Figure S5).

In the reporting-delay model, municipal-hospital records had lower odds of delay >7 days than county/district-hospital records (OR 0.57, 95% CI 0.38–0.86), whereas provincial-hospital records had higher odds (OR 2.45, 95% CI 1.51–4.13). Chest CT documentation (OR 0.54, 95% CI 0.40–0.75) and chest x-ray documentation (OR 0.59, 95% CI 0.40–0.88) were associated with lower odds of delay >7 days (Table 3; Figure 5C). Adjusted probabilities were 78.5% for county/district, 68.0% for municipal, and 89.8% for provincial hospitals; municipal and provincial absolute differences versus county/district hospitals were −10.5 and +11.3 percentage points, respectively (Supplementary Table S8; Supplementary Figure S4). Associations with hospital tier varied across >14- and >30-day thresholds, whereas the inverse association with CT documentation was more consistent (Supplementary Table S7). Excluding extreme delays did not materially change the direction of the primary >7-day associations (Supplementary Table S10).

## Discussion

This analysis identified two prominent and potentially actionable data-quality gaps in a municipal COPD reporting system: incomplete spirometry documentation and delayed system entry. Fewer than one in five records contained any spirometry-related documentation, and only 12.4% contained a valid post-bronchodilator FEV_1_/FVC measurement. Median entry delay was 19 days, and nearly four in five records were entered >7 days after the clinical visit. Although these estimates are specific to the reporting system studied, the underlying quality dimensions—diagnostic-documentation completeness and reporting timeliness—are directly relevant to disease registries and surveillance systems more broadly. These findings concern information available in the reporting system and should not be interpreted as direct estimates of spirometry testing rates, diagnostic accuracy, or population-level case-reporting completeness.

The low level of recorded spirometry is important because GOLD, ATS/ERS, and Chinese guidance emphasize objective airflow assessment for COPD diagnosis (10–12, 33, 38). However, the present data cannot distinguish spirometry that was not performed from spirometry performed previously, at another institution, during another encounter, or simply not transferred into the reporting system. The 19 records with a documented post-bronchodilator FEV_1_/FVC >=0.70 likewise should not be labeled as misdiagnosed, because prior confirmatory testing, clinical stability at testing, and other diagnostic evidence were unavailable (36). This distinction supports interpreting the study as an assessment of documentation quality rather than clinical test utilization.

Older age was associated with absent spirometry documentation, with modest evidence that the association was nonlinear. Potential explanations include difficulty completing technically acceptable maneuvers, acute clinical instability, or reliance on previous diagnostic information; these explanations remain hypotheses because the reporting-system data did not capture reasons for missing spirometry. A useful quality-improvement response would therefore be to document not only spirometry values but also whether testing was completed, deferred, not tolerated, contraindicated, unavailable, or performed elsewhere.

Hospital-tier differences were observed for recorded AECOPD classification, spirometry documentation, and reporting delay. Chinese real-world studies have documented substantial tier-related differences in pulmonary-function testing and COPD treatment patterns (8, 9, 35, 37). In the present study, however, hospital tier may proxy referral patterns, case mix, coding practices, staffing, information systems, and reporting workflows. Individual hospital identifiers and several important clinical confounders were unavailable, so tier coefficients should not be interpreted as independent hospital effects or as causal effects on exacerbation occurrence or reporting quality. Reporting-delay analyses also showed a long right tail and temporal clustering of long-delay entries, particularly among county/district-hospital records. This pattern is compatible with retrospective or batch administrative entry, but the system timestamp did not identify the underlying workflow stage and no workflow metadata were available to confirm the mechanism. The associations of CT and x-ray documentation with lower delay should likewise be viewed as markers of more structured or complete documentation processes rather than evidence that imaging itself improves timeliness. Practical registry indicators could include completeness of post-bronchodilator FEV_1_/FVC fields, documented reasons for unavailable spirometry, and compliance with a clearly defined entry-time standard (21–27).

### Strengths and limitations

This study has several strengths. It used real-world reporting-system data across multiple hospital tiers and evaluated diagnostic-documentation completeness and reporting timeliness within the same surveillance framework. The revision also includes explicit diagnostic-text rules, data-quality checks, alternative outcome definitions, extreme-delay analyses, adjusted absolute probabilities, and model diagnostics in the supplementary material, improving reproducibility. Importantly, the study operationalizes data-quality indicators—spirometry-status completeness and reporting-system entry delay—that can be measured and audited in other COPD registries or reporting systems.

Several limitations should be considered. First, the study was cross-sectional and based on administrative reporting-system data; causal inference is not possible. Second, the source extract did not provide the number of individual contributing hospitals, a denominator of all eligible COPD encounters, or metadata on unsent cases, so representativeness and completeness of case capture cannot be assessed. Third, a separate persistent patient identifier was unavailable; unique reporting-system identifiers therefore do not exclude the possibility of repeated records from the same individual. Fourth, individual hospital identifiers were unavailable, preventing adjustment for within-hospital clustering. Finally, the study was conducted within a single municipal reporting system in Chongqing. Differences in registry design, hospital organization, data-entry workflows, and local reporting practices may limit direct generalizability to other regions or health systems; external validation in other COPD reporting settings is therefore warranted.

Fifth, important clinical variables such as smoking history, biomass exposure, body mass index, symptom scores, comorbidities, medication use, hospitalization status, COPD severity, and longitudinal exacerbation history were unavailable, leaving potential residual confounding. Sixth, AECOPD classification was based on reporting-system diagnosis text rather than independently adjudicated clinical criteria. Seventh, absent spirometry documentation could reflect testing not performed, testing performed but not entered, or prior testing unavailable in the current record. Eighth, the system entry timestamp did not distinguish record creation, submission, approval, or modification, and extreme delays may partly reflect retrospective administrative entry. Ninth, first-diagnosis dates were inconsistent in a minority of records and were not used to estimate diagnostic delay. Finally, findings are limited to Chongqing reporting-system records and may not generalize to other regions or systems.

## Conclusion

Incomplete spirometry documentation and delayed system entry were common in the COPD reporting system evaluated. Older age and hospital tier were associated with absent spirometry documentation, while hospital tier and imaging documentation were associated with >7-day entry delay; several hospital-tier associations varied across sensitivity definitions. These findings highlight practical targets for strengthening COPD surveillance data quality, including structured spirometry-status fields, documented reasons for unavailable testing, explicit reporting-time standards, and audit-feedback mechanisms. Because the study was based on a single municipal system in Chongqing, the magnitude of these gaps should be validated in other regions before broader generalization.

## Data Availability

The data analyzed in this study is subject to the following licenses/restrictions: The patient-level reporting-system data are not publicly available because they are subject to institutional governance and patient privacy restrictions. Access to de-identified analytical data requires a reasonable request to the corresponding author and permission from the data-holding institution. Requests to access these datasets should be directed to Hongying Zhang, Corresponding Author, Affiliated Rehabilitation Hospital of Chongqing Medical University, 900009@hospital.cqmu.edu.cn.
