# Supplementary material for: Spirometry documentation gaps and reporting delays in a COPD reporting system: implications for surveillance data quality
Source: Front Med (Lausanne). 2026 Oct 14;13:1955292. doi: 10.3389/fmed.2026.1955292 (PMC13713816; doi:10.3389/fmed.2026.1955292)
Supplement: Supplementary file 1 [file Supplementaryfile1.docx]

**Spirometry Documentation Gaps and Reporting Delays in a COPD Reporting System: Implications for Surveillance Data Quality**

**Supplementary Materials**

**List of supplementary tables and figures**

Supplementary Table S1. Data-quality and coding checks

Supplementary Table S2. Diagnostic-text expressions and AECOPD classification rules

Supplementary Table S3. Diagnostic documentation by hospital level

Supplementary Table S4. Occupation-group distribution

Supplementary Table S5. Post-bronchodilator fixed-ratio findings

Supplementary Table S6. Sensitivity analysis using a broader AECOPD definition

Supplementary Table S7. Sensitivity analyses using alternative reporting-delay thresholds

Supplementary Table S8. Adjusted predicted probabilities and absolute probability differences for reporting delay >7 days

Supplementary Table S9. Reporting-delay distribution, temporal patterns, and hospital-level patterns

Supplementary Table S10. Sensitivity analyses excluding extreme reporting delays

Supplementary Table S11. Sensitivity analyses for occupation handling

Supplementary Table S12. Descriptive analysis restricted to documented post-bronchodilator FEV1/FVC <0.70

Supplementary Table S13. Regression-model diagnostics and analysis environment

Supplementary Figure S1. Distribution of reporting delay

Supplementary Figure S2. Reporting-system entries by entry month

Supplementary Figure S3. Reporting delay by hospital level

Supplementary Figure S4. Adjusted probability of reporting delay >7 days by hospital level

Supplementary Figure S5. Average adjusted probability of absent spirometry documentation by age

Supplementary File S1. Statistical analysis code (R; plotting code omitted)

**Supplementary Table S1. Data-quality and coding checks**

| **Check** | **n** |
| --- | --- |
| Total raw records | 1109 |
| Age >=40 records | 1076 |
| Duplicate report-card IDs in main cohort | 0 |
| Missing visit date | 0 |
| Missing entry date | 0 |
| Negative reporting delay | 0 |
| Explicit AECOPD classification | 357 |
| Broad AECOPD classification | 488 |
| Acute lower respiratory infection label | 131 |
| Pneumonia label | 0 |
| Respiratory failure label | 0 |
| Any spirometry documentation | 195 |
| Pre-BD raw -1 sentinel | 65 |
| Post-BD raw -1 sentinel | 72 |
| Pre-BD FEV1/FVC valid/recorded after sentinel removal | 143 |
| Post-BD FEV1/FVC valid/recorded after sentinel removal | 133 |
| Post-BD FEV1/FVC <70 among recorded | 114 |

**Note:** The main cohort contained 1,076 records aged 40 years or older. Raw FEV1/FVC values coded as -1 were treated as missing sentinel values before determining whether quantitative spirometry values were recorded. Report-card identifiers were unique, but this does not establish unique patients in the absence of a separate patient identifier.

**Supplementary Table S2. Diagnostic-text expressions and AECOPD classification rules**

| **Original diagnosis text** | **n** | **Primary AECOPD** | **Acute lower respiratory infection** | **Broad sensitivity AECOPD** |
| --- | --- | --- | --- | --- |
| Chronic obstructive pulmonary disease, unspecified | 374 | No | No | No |
| Chronic obstructive pulmonary disease with acute exacerbation, unspecified | 357 | Yes | No | Yes |
| Chronic obstructive pulmonary disease with acute lower respiratory infection | 131 | No | Yes | Yes |
| Other specified chronic obstructive pulmonary disease | 104 | No | No | No |
| Other chronic obstructive pulmonary disease | 96 | No | No | No |
| Asthma | 6 | No | No | No |
| Asthma, unspecified | 3 | No | No | No |
| Emphysema, unspecified | 3 | No | No | No |
| J40 Bronchitis, not specified as acute or chronic | 1 | No | No | No |
| Unspecified chronic bronchitis | 1 | No | No | No |

**Note:** The primary AECOPD definition required explicit acute-exacerbation wording. The broader sensitivity definition additionally classified records labeled as COPD with acute lower respiratory tract infection as AECOPD. No diagnosis-text records were flagged for pneumonia or respiratory failure by the prespecified text rules.

**Supplementary Table S3. Diagnostic documentation by hospital level**

| **Hospital level** | **N** | **Clinical symptoms** | **Any spirometry** | **Spirometry as basis** | **Pre-BD FEV1/FVC** | **Post-BD FEV1/FVC** | **Chest CT** | **Chest X-ray** | **Laboratory tests** |
| --- | --- | --- | --- | --- | --- | --- | --- | --- | --- |
| County/district hospital | 678 | 618 (91.2%) | 119 (17.6%) | 97 (14.3%) | 80 (11.8%) | 76 (11.2%) | 256 (37.8%) | 101 (14.9%) | 119 (17.6%) |
| Township hospital | 51 | 41 (80.4%) | 10 (19.6%) | 10 (19.6%) | 7 (13.7%) | 7 (13.7%) | 8 (15.7%) | 20 (39.2%) | 10 (19.6%) |
| Municipal hospital | 152 | 104 (68.4%) | 36 (23.7%) | 31 (20.4%) | 30 (19.7%) | 31 (20.4%) | 79 (52.0%) | 8 (5.3%) | 18 (11.8%) |
| Provincial hospital | 191 | 138 (72.3%) | 29 (15.2%) | 28 (14.7%) | 25 (13.1%) | 18 (9.4%) | 103 (53.9%) | 69 (36.1%) | 28 (14.7%) |
| Other | 4 | 3 (75.0%) | 1 (25.0%) | 1 (25.0%) | 1 (25.0%) | 1 (25.0%) | 0 (0.0%) | 0 (0.0%) | 0 (0.0%) |

**Note:** Values are n (%). The sparse 'Other' hospital-level category (n=4) is shown descriptively but was excluded from multivariable models.

**Supplementary Table S4. Occupation-group distribution**

| **Occupation group** | **n** | **%** |
| --- | --- | --- |
| Other/uncategorized | 770 | 71.6% |
| Agriculture/forestry/fishery | 63 | 5.9% |
| Unemployed | 150 | 13.9% |
| Other occupations | 93 | 8.6% |

**Note:** Occupation groups were derived from the original industry and specific-occupation fields. The other/uncategorized group remained large and heterogeneous; occupation-related model estimates were therefore treated as exploratory.

**Supplementary Table S5. Post-bronchodilator fixed-ratio findings**

| **Recorded post-BD FEV1/FVC status** | **n** | **% of records with post-BD value** |
| --- | --- | --- |
| Post-BD FEV1/FVC <70% | 114 | 85.7% |
| Post-BD FEV1/FVC ≥70% | 19 | 14.3% |

**Note:** A total of 133 records contained a valid post-bronchodilator FEV1/FVC value after removal of -1 sentinel values. The reporting-system data could not determine whether patients with values ≥0.70 had prior confirmatory spirometry, testing during clinical stability, or other evidence supporting the registered COPD diagnosis.

**Supplementary Table S6. Sensitivity analysis using a broader AECOPD definition**

| **Variable** | **OR (95% CI)** | **P value** |
| --- | --- | --- |
| Age, per year | 1.01 (1.00-1.02) | 0.110 |
| Female vs male | 0.92 (0.69-1.24) | 0.598 |
| Agriculture/forestry/fishery vs other/uncategorized | 1.22 (0.66-2.27) | 0.523 |
| Unemployed vs other/uncategorized | 0.99 (0.67-1.45) | 0.944 |
| Other occupations vs other/uncategorized | 0.91 (0.57-1.42) | 0.672 |
| Township hospital vs county/district hospital | 1.44 (0.74-2.81) | 0.277 |
| Municipal hospital vs county/district hospital | 0.48 (0.32-0.70) | <0.001 |
| Provincial hospital vs county/district hospital | 0.79 (0.56-1.11) | 0.178 |
| Spirometry documented vs not documented | 1.21 (0.87-1.68) | 0.267 |
| Chest CT documented vs not documented | 1.99 (1.53-2.59) | <0.001 |
| Chest X-ray documented vs not documented | 1.40 (1.00-1.97) | 0.048 |
| Reporting delay >7 days vs ≤7 days | 1.07 (0.79-1.47) | 0.661 |

**Note:** The broader AECOPD definition included records labeled as COPD with acute lower respiratory tract infection in addition to records with explicit acute-exacerbation wording. All prespecified covariates were entered simultaneously.

**Supplementary Table S7. Sensitivity analyses using alternative reporting-delay thresholds**

| **Variable** | **>7 d OR (95% CI)** | **P** | **>14 d OR (95% CI)** | **P** | **>30 d OR (95% CI)** | **P** |
| --- | --- | --- | --- | --- | --- | --- |
| Age, per year | 1.00 (0.98-1.01) | 0.749 | 1.00 (0.98-1.01) | 0.569 | 0.99 (0.97-1.00) | 0.087 |
| Female vs male | 0.81 (0.57-1.15) | 0.228 | 0.84 (0.62-1.13) | 0.245 | 1.11 (0.79-1.55) | 0.531 |
| Agriculture/forestry/fishery vs other/uncategorized | 1.31 (0.64-2.89) | 0.476 | 1.81 (0.94-3.62) | 0.083 | 1.35 (0.70-2.58) | 0.364 |
| Unemployed vs other/uncategorized | 1.16 (0.73-1.89) | 0.529 | 1.02 (0.70-1.52) | 0.906 | 0.50 (0.29-0.83) | 0.009 |
| Other occupations vs other/uncategorized | 0.98 (0.58-1.72) | 0.938 | 0.85 (0.54-1.34) | 0.475 | 0.88 (0.52-1.46) | 0.629 |
| Township hospital vs county/district hospital | 0.69 (0.32-1.56) | 0.355 | 0.47 (0.24-0.93) | 0.031 | 0.76 (0.36-1.54) | 0.449 |
| Municipal hospital vs county/district hospital | 0.57 (0.38-0.86) | 0.007 | 0.39 (0.27-0.57) | <0.001 | 0.48 (0.29-0.76) | 0.002 |
| Provincial hospital vs county/district hospital | 2.45 (1.51-4.13) | <0.001 | 1.41 (0.98-2.05) | 0.065 | 0.44 (0.28-0.68) | <0.001 |
| Spirometry documented vs not documented | 0.88 (0.60-1.32) | 0.529 | 1.01 (0.72-1.42) | 0.975 | 0.87 (0.58-1.27) | 0.471 |
| Chest CT documented vs not documented | 0.54 (0.40-0.75) | <0.001 | 0.53 (0.41-0.69) | <0.001 | 0.47 (0.34-0.64) | <0.001 |
| Chest X-ray documented vs not documented | 0.59 (0.40-0.88) | 0.010 | 0.88 (0.62-1.24) | 0.453 | 1.41 (0.96-2.05) | 0.073 |
| AECOPD vs non-AECOPD | 1.20 (0.86-1.68) | 0.282 | 1.18 (0.90-1.57) | 0.239 | 1.21 (0.90-1.64) | 0.205 |

**Note:** The >7-day threshold was the primary outcome; >14-day and >30-day thresholds were sensitivity analyses. Hospital-tier associations varied across thresholds, whereas the association with chest CT documentation was directionally consistent.

**Supplementary Table S8. Adjusted predicted probabilities and absolute probability differences for reporting delay >7 days**

| **Group** | **Adjusted probability** | **95% CI** | **Absolute difference, percentage points** | **95% CI of difference** | **P value** |
| --- | --- | --- | --- | --- | --- |
| County/district hospital | 78.5% | 75.4-81.7% |  |  |  |
| Township hospital | 71.9% | 57.1-86.7% | -6.7 | -21.8 to 8.5 | 0.390 |
| Municipal hospital | 68.0% | 60.5-75.5% | -10.5 | -18.7 to -2.3 | 0.012 |
| Provincial hospital | 89.8% | 85.7-93.9% | 11.3 | 6.0 to 16.5 | <0.001 |
| Chest CT not documented | 83.0% | 80.1-86.0% |  |  |  |
| Chest CT documented | 73.1% | 68.8-77.3% | -10.0 | -15.2 to -4.7 | <0.001 |
| Chest X-ray not documented | 80.5% | 77.9-83.1% |  |  |  |
| Chest X-ray documented | 71.5% | 64.7-78.2% | -9.0 | -16.4 to -1.7 | 0.015 |

**Note:** Hospital-level absolute differences use county/district hospitals as the reference. Imaging differences compare documented with not documented. Probabilities and contrasts are model-based marginal estimates; associations should not be interpreted causally.

**Supplementary Table S9. Reporting-delay distribution, temporal patterns, and hospital-level patterns**

**Panel A. Distribution and extreme-delay frequencies**

| **Measure** | **Value** |
| --- | --- |
| Minimum | 0.00 days |
| 1st percentile | 0.00 days |
| 5th percentile | 0.00 days |
| 25th percentile | 9.00 days |
| 50th percentile | 19.00 days |
| 75th percentile | 32.25 days |
| 90th percentile | 122.50 days |
| 95th percentile | 232.50 days |
| 99th percentile | 264.00 days |
| Maximum | 325.00 days |
| >90 days | 123 (11.4%) |
| >180 days | 79 (7.3%) |
| >99th percentile | 10 (0.9%) |
| >200 days | 74 (6.9%) |

**Panel B. Reporting delay by visit month**

| **Visit month** | **n** | **Median, d** | **IQR, d** | **>7 d** | **>14 d** | **>30 d** |
| --- | --- | --- | --- | --- | --- | --- |
| 2024-01 | 229 | 20.0 | 9.0-45.0 | 77.3% | 65.5% | 32.3% |
| 2024-02 | 144 | 20.0 | 11.0-36.2 | 83.3% | 67.4% | 29.2% |
| 2024-03 | 117 | 17.0 | 8.0-33.0 | 75.2% | 55.6% | 26.5% |
| 2024-04 | 83 | 16.0 | 10.0-34.5 | 81.9% | 56.6% | 27.7% |
| 2024-05 | 79 | 17.0 | 8.0-40.5 | 77.2% | 55.7% | 31.6% |
| 2024-06 | 66 | 19.0 | 13.0-55.2 | 84.8% | 66.7% | 36.4% |
| 2024-07 | 75 | 21.0 | 12.0-34.5 | 80.0% | 64.0% | 28.0% |
| 2024-08 | 49 | 18.0 | 6.0-35.0 | 69.4% | 55.1% | 28.6% |
| 2024-09 | 38 | 15.5 | 7.0-26.0 | 71.1% | 52.6% | 15.8% |
| 2024-10 | 64 | 18.0 | 9.0-26.2 | 79.7% | 57.8% | 15.6% |
| 2024-11 | 73 | 20.0 | 10.0-28.0 | 87.7% | 65.8% | 16.4% |
| 2024-12 | 59 | 15.0 | 8.0-23.0 | 76.3% | 52.5% | 11.9% |

**Panel C. Reporting-system entries by entry month**

| **Entry month** | **Records entered** | **Median delay, d** | **IQR, d** |
| --- | --- | --- | --- |
| 2024-01 | 83 | 7.0 | 2.0-14.0 |
| 2024-02 | 154 | 18.0 | 11.0-25.0 |
| 2024-03 | 103 | 16.0 | 6.5-26.0 |
| 2024-04 | 91 | 20.0 | 10.0-30.5 |
| 2024-05 | 73 | 13.0 | 7.0-25.0 |
| 2024-06 | 57 | 20.0 | 14.0-36.0 |
| 2024-07 | 75 | 15.0 | 9.0-29.5 |
| 2024-08 | 56 | 21.0 | 6.0-30.0 |
| 2024-09 | 160 | 162.5 | 47.8-240.5 |
| 2024-10 | 42 | 12.5 | 7.0-23.0 |
| 2024-11 | 63 | 19.0 | 10.0-27.0 |
| 2024-12 | 79 | 20.0 | 9.5-28.5 |
| 2025-01 | 40 | 23.0 | 15.8-32.0 |

**Panel D. Reporting delay by hospital level**

| **Hospital level** | **N** | **Median, d** | **IQR, d** | **>7 d** | **>14 d** | **>30 d** | **>90 d** |
| --- | --- | --- | --- | --- | --- | --- | --- |
| County/district hospital | 678 | 21.0 | 9.0-42.8 | 538 (79.4%) | 436 (64.3%) | 217 (32.0%) | 116 (17.1%) |
| Township hospital | 51 | 18.0 | 8.0-42.5 | 39 (76.5%) | 29 (56.9%) | 17 (33.3%) | 6 (11.8%) |
| Municipal hospital | 152 | 11.0 | 6.0-22.2 | 102 (67.1%) | 60 (39.5%) | 24 (15.8%) | 0 (0.0%) |
| Provincial hospital | 191 | 18.0 | 13.0-27.0 | 168 (88.0%) | 130 (68.1%) | 30 (15.7%) | 1 (0.5%) |
| Other | 4 | 22.0 | 14.2-35.0 | 4 (100.0%) | 3 (75.0%) | 1 (25.0%) | 0 (0.0%) |

**Note:** A marked concentration of long-delay entries was observed among records entered in September 2024 (n=160; median delay 162.5 days, IQR 47.75-240.5). This temporal pattern is compatible with retrospective or batch entry, but the reporting-system extract did not contain workflow metadata to confirm the mechanism. The de-identified analytical extract used for this study was generated and transferred between October and December 2025.

**Supplementary Table S10. Sensitivity analyses excluding extreme reporting delays**

| **Variable** | **Exclude >99th percentile OR (95% CI)** | **P** | **Exclude >180 d OR (95% CI)** | **P** |
| --- | --- | --- | --- | --- |
| Age, per year | 1.00 (0.98-1.01) | 0.766 | 1.00 (0.98-1.01) | 0.882 |
| Female vs male | 0.81 (0.57-1.15) | 0.230 | 0.81 (0.57-1.16) | 0.244 |
| Agriculture/forestry/fishery vs other/uncategorized | 1.35 (0.65-2.98) | 0.434 | 1.38 (0.67-3.04) | 0.396 |
| Unemployed vs other/uncategorized | 1.18 (0.74-1.91) | 0.499 | 1.21 (0.77-1.96) | 0.418 |
| Other occupations vs other/uncategorized | 0.99 (0.59-1.74) | 0.979 | 1.08 (0.64-1.88) | 0.788 |
| Township hospital vs county/district hospital | 0.68 (0.32-1.55) | 0.341 | 0.77 (0.36-1.73) | 0.508 |
| Municipal hospital vs county/district hospital | 0.58 (0.39-0.87) | 0.008 | 0.65 (0.43-0.98) | 0.036 |
| Provincial hospital vs county/district hospital | 2.48 (1.53-4.18) | <0.001 | 2.71 (1.67-4.56) | <0.001 |
| Spirometry documented vs not documented | 0.89 (0.60-1.33) | 0.563 | 0.96 (0.66-1.44) | 0.856 |
| Chest CT documented vs not documented | 0.55 (0.40-0.76) | <0.001 | 0.61 (0.45-0.84) | 0.002 |
| Chest X-ray documented vs not documented | 0.59 (0.40-0.88) | 0.009 | 0.62 (0.42-0.93) | 0.019 |
| AECOPD vs non-AECOPD | 1.19 (0.86-1.67) | 0.302 | 1.17 (0.84-1.65) | 0.346 |

**Note:** The 99th percentile of reporting delay was 264 days. Excluding extreme delays did not materially alter the direction of the principal >7-day reporting-delay associations.

**Supplementary Table S11. Sensitivity analyses for occupation handling**

| **Variable** | **No-spirometry model: occupation omitted OR (95% CI)** | **P** | **Delay >7 d model: occupation omitted OR (95% CI)** | **P** | **No-spirometry model: missing occupation excluded OR (95% CI)** | **P** |
| --- | --- | --- | --- | --- | --- | --- |
| Age, per year | 1.03 (1.01-1.04) | 0.003 | 1.00 (0.98-1.01) | 0.753 | 1.03 (1.01-1.04) | 0.002 |
| Female vs male | 0.75 (0.52-1.08) | 0.120 | 0.81 (0.57-1.15) | 0.233 | 0.79 (0.55-1.15) | 0.216 |
| Township hospital vs county/district hospital | 0.91 (0.45-2.00) | 0.805 | 0.80 (0.41-1.66) | 0.522 | 1.51 (0.69-3.56) | 0.320 |
| Municipal hospital vs county/district hospital | 0.63 (0.41-0.99) | 0.041 | 0.57 (0.38-0.85) | 0.005 | 0.52 (0.33-0.82) | 0.005 |
| Provincial hospital vs county/district hospital | 1.30 (0.82-2.09) | 0.274 | 2.46 (1.52-4.14) | <0.001 | 1.30 (0.81-2.14) | 0.296 |
| Chest CT documented vs not documented | 0.93 (0.67-1.29) | 0.668 | 0.55 (0.41-0.75) | <0.001 | 1.19 (0.84-1.69) | 0.321 |
| Chest X-ray documented vs not documented | 0.65 (0.44-0.98) | 0.036 | 0.61 (0.42-0.91) | 0.014 | 0.74 (0.49-1.15) | 0.172 |
| AECOPD vs non-AECOPD | 1.06 (0.75-1.50) | 0.750 | 1.21 (0.87-1.69) | 0.266 | 1.06 (0.75-1.52) | 0.739 |

**Note:** Occupation-related estimates were not emphasized because the other/uncategorized reference category was large and heterogeneous. The principal age and hospital-level findings were not materially changed by alternative occupation handling.

**Supplementary Table S12. Descriptive analysis restricted to documented post-bronchodilator FEV1/FVC <0.70**

| **Subgroup** | **n** | **AECOPD** | **Delay >7 days** | **Reporting delay, median (IQR), d** |
| --- | --- | --- | --- | --- |
| Post-BD FEV1/FVC <0.70 | 113 | 43 (38.1%) | 83 (73.5%) | 17.0 (7.0-28.0) |

**Note:** The model-analysis subgroup contained 113 records because sparse unknown-sex and 'Other' hospital-level categories were excluded from multivariable modeling. Exploratory multivariable models in this subgroup were unstable because of small sample size and sparse hospital-level strata; subgroup findings are therefore presented descriptively.

**Supplementary Table S13. Regression-model diagnostics and analysis environment**

| **Outcome** | **Model N** | **Events** | **Events/parameter** | **Spline vs linear age LRT P** | **Maximum VIF** | **Cook's D >4/n** |
| --- | --- | --- | --- | --- | --- | --- |
| AECOPD explicit | 1071 | 354 | 27.2 | 0.218 | 1.56 | 34 (3.2%) |
| No spirometry documentation | 1071 | 878 | 73.2 | 0.031 | 1.52 | 86 (8.0%) |
| Reporting delay >7 days | 1071 | 846 | 65.1 | 0.571 | 1.62 | 79 (7.4%) |

**Note:** Only the no-spirometry-documentation model showed evidence that a 3-df natural cubic spline improved fit over a linear age term (LRT P=0.031). All VIFs were <2. Cook's distance was used diagnostically; wholesale exclusion of observations above the heuristic 4/n threshold caused sparse-data instability in some categorical strata and was not used as the primary basis for case deletion. Individual hospital identifiers were unavailable, so within-hospital clustering could not be modeled. Analyses were conducted in R version 4.5.2 on Windows 11. The complete statistical analysis script used for the reported analyses is provided as Supplementary File S1; plotting code is intentionally omitted.

**Supplementary Figure S1. Distribution of reporting delay**

**Note:** Reporting delay was defined as reporting-system entry date minus clinical visit date. Dashed vertical lines indicate 7-, 14-, and 30-day thresholds. Values above the 99th percentile (264 days) were omitted for graphical display only; all valid observations were retained in the primary analyses.

**Supplementary Figure S2. Reporting-system entries by entry month**

**Note:** Monthly record-entry counts were examined to assess temporal clustering of reporting activity and possible batch-entry patterns. A marked entry peak occurred in September 2024.

**Supplementary Figure S3. Reporting delay by hospital level**

**Note:** Boxplots show reporting-delay distributions by hospital level. The y-axis is truncated at the overall 99th percentile for display. The sparse 'Other' hospital category is omitted from the figure.

**Supplementary Figure S4. Adjusted probability of reporting delay >7 days by hospital level**

**Note:** Points show model-based adjusted predicted probabilities and error bars show 95% confidence intervals. Predictions were obtained from the primary multivariable logistic regression model.

**Supplementary Figure S5. Average adjusted probability of absent spirometry documentation by age**

**Note:** Marginal predictions from a natural cubic spline model with 3 degrees of freedom, standardized over the observed covariate distribution. Shading shows 95% confidence intervals, truncated to the probability range 0-1 for display. Uncertainty is wider at the extremes of the age distribution.

**Supplementary File S1. Statistical analysis code (R; plotting code omitted)**

This script reproduces the statistical analyses reported in the manuscript and supplementary tables. Because the municipal reporting platform uses system-specific source-field labels, the shared script begins from a de-identified analysis-ready dataset with standardized variable names mapped according to Supplementary Tables S1-S5. No plotting code is included.

```
# Supplementary File S1. Statistical analysis code
# Manuscript: Spirometry Documentation Gaps and Reporting Delays in a COPD
# Reporting System: Implications for Surveillance Data Quality
#
# Analysis environment reported in the manuscript:
# R version 4.5.2, Windows 11
#
# IMPORTANT:
# This script starts from the de-identified, analysis-ready extract with
# standardized variable names. Raw source-field labels in the municipal
# reporting system were mapped locally to these names according to the
# definitions in Supplementary Tables S1-S5.
#
# Plotting code is intentionally omitted.

# ============================================================
# 1. Packages
# ============================================================

required_packages <- c(
  "dplyr",
  "tidyr",
  "stringr",
  "lubridate",
  "broom",
  "car",
  "splines",
  "marginaleffects"
)

to_install <- required_packages[
  !required_packages %in% rownames(installed.packages())
]

if (length(to_install) > 0) {
  install.packages(to_install)
}

library(dplyr)
library(tidyr)
library(stringr)
library(lubridate)
library(broom)
library(car)
library(splines)
library(marginaleffects)


# ============================================================
# 2. Import de-identified analysis-ready data
# ============================================================
#
# Expected standardized variables:
#
# report_id
# birth_date
# visit_date
# entry_datetime
# sex
# ethnicity
# marital_status
# occupation_group
# hospital_level
# diagnosis_text
# diagnostic_basis_text
# pre_bd_fev1_fvc_raw
# post_bd_fev1_fvc_raw
# death_recorded
#
# Example import:
#
# dat_raw <- read.csv(
#   "copd_reporting_analysis.csv",
#   stringsAsFactors = FALSE,
#   check.names = FALSE
# )
#
# In the study dataset, the de-identified extract was generated/transferred
# between October and December 2025 and contained records with clinical
# visit dates in 2024.

stopifnot(exists("dat_raw"))


# ============================================================
# 3. Date handling, age, and reporting delay
# ============================================================

dat <- dat_raw %>%
  mutate(
    birth_date = as.Date(birth_date),
    visit_date = as.Date(visit_date),
    entry_date = as.Date(entry_datetime),

    age = as.numeric(
      difftime(visit_date, birth_date, units = "days")
    ) / 365.25,

    reporting_delay = as.numeric(
      entry_date - visit_date
    )
  )


# ============================================================
# 4. Main cohort and data-quality checks
# ============================================================

main <- dat %>%
  filter(
    !is.na(age),
    age >= 40
  )

# Core checks reported in Supplementary Table S1
nrow(dat)                             # expected raw n = 1109
nrow(main)                            # expected age >=40 n = 1076
sum(duplicated(main$report_id))       # expected 0
sum(is.na(main$visit_date))           # expected 0
sum(is.na(main$entry_date))           # expected 0
sum(main$reporting_delay < 0, na.rm = TRUE)  # expected 0


# ============================================================
# 5. Spirometry variable handling
# ============================================================

main <- main %>%
  mutate(
    # -1 was the sentinel for unavailable numeric measurements
    pre_bd_fev1_fvc = ifelse(
      pre_bd_fev1_fvc_raw == -1,
      NA_real_,
      as.numeric(pre_bd_fev1_fvc_raw)
    ),

    post_bd_fev1_fvc = ifelse(
      post_bd_fev1_fvc_raw == -1,
      NA_real_,
      as.numeric(post_bd_fev1_fvc_raw)
    ),

    pre_bd_recorded = !is.na(pre_bd_fev1_fvc),
    post_bd_recorded = !is.na(post_bd_fev1_fvc),

    spirometry_basis = str_detect(
      str_to_lower(coalesce(diagnostic_basis_text, "")),
      "pulmonary function|spirometry"
    ),

    any_spirometry = (
      spirometry_basis |
      pre_bd_recorded |
      post_bd_recorded
    ),

    absent_spirometry = !any_spirometry,

    post_bd_obstruction = case_when(
      is.na(post_bd_fev1_fvc) ~ NA,
      post_bd_fev1_fvc < 70 ~ TRUE,
      TRUE ~ FALSE
    )
  )


# ============================================================
# 6. AECOPD classification
# ============================================================
#
# Primary definition:
# explicit acute-exacerbation wording.
#
# Broad sensitivity definition:
# primary definition OR COPD with acute lower respiratory infection.
#
# The exact source-text dictionary is provided in Supplementary Table S2.

main <- main %>%
  mutate(
    dx_norm = str_squish(
      str_to_lower(coalesce(diagnosis_text, ""))
    ),

    aecopd_explicit = str_detect(
      dx_norm,
      "chronic obstructive pulmonary disease with acute exacerbation"
    ),

    acute_lri = str_detect(
      dx_norm,
      "chronic obstructive pulmonary disease with acute lower respiratory infection"
    ),

    aecopd_broad = aecopd_explicit | acute_lri
  )


# ============================================================
# 7. Diagnostic-documentation indicators
# ============================================================

main <- main %>%
  mutate(
    basis_norm = str_to_lower(
      coalesce(diagnostic_basis_text, "")
    ),

    clinical_symptoms = str_detect(
      basis_norm,
      "clinical symptom"
    ),

    chest_ct = str_detect(
      basis_norm,
      "ct|computed tomography"
    ),

    chest_xray = str_detect(
      basis_norm,
      "x-ray|x ray|radiograph"
    ),

    laboratory_tests = str_detect(
      basis_norm,
      "laboratory|lab test"
    )
  )


# ============================================================
# 8. Reporting-delay outcomes
# ============================================================

main <- main %>%
  mutate(
    delay_gt7  = reporting_delay > 7,
    delay_gt14 = reporting_delay > 14,
    delay_gt30 = reporting_delay > 30,
    delay_gt90 = reporting_delay > 90,
    delay_gt180 = reporting_delay > 180
  )


# ============================================================
# 9. Factor coding and model-eligible dataset
# ============================================================

main <- main %>%
  mutate(
    sex = factor(sex),
    occupation_group = factor(
      occupation_group,
      levels = c(
        "Other/uncategorized",
        "Agriculture/forestry/fishery",
        "Unemployed",
        "Other occupations"
      )
    ),
    hospital_level = factor(
      hospital_level,
      levels = c(
        "County/district hospital",
        "Township hospital",
        "Municipal hospital",
        "Provincial hospital",
        "Other"
      )
    )
  )

# Primary multivariable models excluded:
# 1 record with unknown sex
# 4 records in the sparse "Other" hospital-level category

model_dat <- main %>%
  filter(
    !is.na(sex),
    sex %in% c("Male", "Female"),
    hospital_level != "Other"
  ) %>%
  droplevels()

nrow(model_dat)  # expected n = 1071


# ============================================================
# 10. Descriptive statistics
# ============================================================

# Age
median(main$age, na.rm = TRUE)
quantile(main$age, probs = c(0.25, 0.75), na.rm = TRUE)

# AECOPD
sum(main$aecopd_explicit, na.rm = TRUE)
mean(main$aecopd_explicit, na.rm = TRUE)

# Spirometry documentation
sum(main$any_spirometry, na.rm = TRUE)
mean(main$any_spirometry, na.rm = TRUE)

sum(main$spirometry_basis, na.rm = TRUE)
mean(main$spirometry_basis, na.rm = TRUE)

sum(main$post_bd_recorded, na.rm = TRUE)
mean(main$post_bd_recorded, na.rm = TRUE)

# Fixed-ratio findings among records with a valid post-BD value
main %>%
  filter(post_bd_recorded) %>%
  summarise(
    n = n(),
    lt_070 = sum(post_bd_fev1_fvc < 70),
    ge_070 = sum(post_bd_fev1_fvc >= 70),
    pct_lt_070 = mean(post_bd_fev1_fvc < 70) * 100
  )

# Reporting delay
median(main$reporting_delay, na.rm = TRUE)
quantile(
  main$reporting_delay,
  probs = c(0.25, 0.75, 0.90, 0.95, 0.99),
  na.rm = TRUE
)

sapply(
  c(7, 14, 30, 90, 180, 200),
  function(x) {
    c(
      n = sum(main$reporting_delay > x, na.rm = TRUE),
      pct = mean(main$reporting_delay > x, na.rm = TRUE) * 100
    )
  }
)


# ============================================================
# 11. Primary multivariable logistic-regression models
# ============================================================

common_covariates <- paste(
  "age + sex + occupation_group + hospital_level +",
  "any_spirometry + chest_ct + chest_xray"
)

# Model A: recorded AECOPD classification
fit_aecopd <- glm(
  aecopd_explicit ~
    age +
    sex +
    occupation_group +
    hospital_level +
    any_spirometry +
    chest_ct +
    chest_xray +
    delay_gt7,
  data = model_dat,
  family = binomial()
)

# Model B: absent spirometry documentation
fit_no_spiro <- glm(
  absent_spirometry ~
    age +
    sex +
    occupation_group +
    hospital_level +
    chest_ct +
    chest_xray +
    aecopd_explicit,
  data = model_dat,
  family = binomial()
)

# Model C: reporting delay >7 days
fit_delay7 <- glm(
  delay_gt7 ~
    age +
    sex +
    occupation_group +
    hospital_level +
    any_spirometry +
    chest_ct +
    chest_xray +
    aecopd_explicit,
  data = model_dat,
  family = binomial()
)


# ============================================================
# 12. Odds ratios, confidence intervals, and P values
# ============================================================

or_table <- function(model) {
  broom::tidy(
    model,
    exponentiate = TRUE,
    conf.int = TRUE
  ) %>%
    transmute(
      term,
      OR = estimate,
      lower_95CI = conf.low,
      upper_95CI = conf.high,
      p_value = p.value
    )
}

aecopd_results <- or_table(fit_aecopd)
no_spiro_results <- or_table(fit_no_spiro)
delay7_results <- or_table(fit_delay7)

aecopd_results
no_spiro_results
delay7_results


# ============================================================
# 13. Model sample sizes and event counts
# ============================================================

model_summary <- tibble(
  outcome = c(
    "AECOPD explicit",
    "No spirometry documentation",
    "Reporting delay >7 days"
  ),
  N = c(
    nobs(fit_aecopd),
    nobs(fit_no_spiro),
    nobs(fit_delay7)
  ),
  events = c(
    sum(model.response(model.frame(fit_aecopd)) == 1),
    sum(model.response(model.frame(fit_no_spiro)) == 1),
    sum(model.response(model.frame(fit_delay7)) == 1)
  )
) %>%
  mutate(
    parameters = c(
      length(coef(fit_aecopd)) - 1,
      length(coef(fit_no_spiro)) - 1,
      length(coef(fit_delay7)) - 1
    ),
    events_per_parameter = events / parameters
  )

model_summary


# ============================================================
# 14. Multicollinearity diagnostics
# ============================================================

vif_aecopd <- car::vif(fit_aecopd)
vif_no_spiro <- car::vif(fit_no_spiro)
vif_delay7 <- car::vif(fit_delay7)

vif_aecopd
vif_no_spiro
vif_delay7


# ============================================================
# 15. Influence diagnostics
# ============================================================

cook_summary <- function(model) {
  cd <- cooks.distance(model)
  cutoff <- 4 / length(cd)

  tibble(
    n = length(cd),
    cutoff_4_over_n = cutoff,
    n_above_cutoff = sum(cd > cutoff, na.rm = TRUE),
    pct_above_cutoff = mean(cd > cutoff, na.rm = TRUE) * 100,
    max_cooks_d = max(cd, na.rm = TRUE)
  )
}

cook_aecopd <- cook_summary(fit_aecopd)
cook_no_spiro <- cook_summary(fit_no_spiro)
cook_delay7 <- cook_summary(fit_delay7)

cook_aecopd
cook_no_spiro
cook_delay7


# ============================================================
# 16. Age-linearity assessment using natural cubic splines
# ============================================================

fit_aecopd_spline <- update(
  fit_aecopd,
  . ~ . - age + ns(age, df = 3)
)

fit_no_spiro_spline <- update(
  fit_no_spiro,
  . ~ . - age + ns(age, df = 3)
)

fit_delay7_spline <- update(
  fit_delay7,
  . ~ . - age + ns(age, df = 3)
)

anova(
  fit_aecopd,
  fit_aecopd_spline,
  test = "LRT"
)

anova(
  fit_no_spiro,
  fit_no_spiro_spline,
  test = "LRT"
)

anova(
  fit_delay7,
  fit_delay7_spline,
  test = "LRT"
)


# ============================================================
# 17. Broader AECOPD sensitivity definition
# ============================================================

fit_aecopd_broad <- glm(
  aecopd_broad ~
    age +
    sex +
    occupation_group +
    hospital_level +
    any_spirometry +
    chest_ct +
    chest_xray +
    delay_gt7,
  data = model_dat,
  family = binomial()
)

aecopd_broad_results <- or_table(
  fit_aecopd_broad
)

aecopd_broad_results


# ============================================================
# 18. Alternative reporting-delay thresholds
# ============================================================

fit_delay14 <- glm(
  delay_gt14 ~
    age +
    sex +
    occupation_group +
    hospital_level +
    any_spirometry +
    chest_ct +
    chest_xray +
    aecopd_explicit,
  data = model_dat,
  family = binomial()
)

fit_delay30 <- glm(
  delay_gt30 ~
    age +
    sex +
    occupation_group +
    hospital_level +
    any_spirometry +
    chest_ct +
    chest_xray +
    aecopd_explicit,
  data = model_dat,
  family = binomial()
)

delay14_results <- or_table(fit_delay14)
delay30_results <- or_table(fit_delay30)

delay14_results
delay30_results


# ============================================================
# 19. Adjusted predicted probabilities and absolute differences
#     for reporting delay >7 days
# ============================================================

# Hospital-level adjusted probabilities
avg_predictions(
  fit_delay7,
  newdata = datagrid(model = fit_delay7, hospital_level = levels(model_dat$hospital_level), grid_type = "counterfactual"),
  type = "response"
)

# Pairwise hospital-level differences
avg_comparisons(
  fit_delay7,
  variables = "hospital_level",
  type = "response"
)

# Chest CT adjusted probabilities and difference
avg_predictions(
  fit_delay7,
  newdata = datagrid(model = fit_delay7, chest_ct = c(FALSE, TRUE), grid_type = "counterfactual"),
  type = "response"
)

avg_comparisons(
  fit_delay7,
  variables = "chest_ct",
  type = "response"
)

# Chest X-ray adjusted probabilities and difference
avg_predictions(
  fit_delay7,
  newdata = datagrid(model = fit_delay7, chest_xray = c(FALSE, TRUE), grid_type = "counterfactual"),
  type = "response"
)

avg_comparisons(
  fit_delay7,
  variables = "chest_xray",
  type = "response"
)


# ============================================================
# 20. Reporting-delay temporal analyses
# ============================================================

delay_by_visit_month <- main %>%
  mutate(
    visit_month = format(visit_date, "%Y-%m")
  ) %>%
  group_by(visit_month) %>%
  summarise(
    n = n(),
    median_delay = median(reporting_delay, na.rm = TRUE),
    q1 = quantile(reporting_delay, 0.25, na.rm = TRUE),
    q3 = quantile(reporting_delay, 0.75, na.rm = TRUE),
    pct_gt7 = mean(delay_gt7, na.rm = TRUE) * 100,
    pct_gt14 = mean(delay_gt14, na.rm = TRUE) * 100,
    pct_gt30 = mean(delay_gt30, na.rm = TRUE) * 100,
    .groups = "drop"
  )

delay_by_entry_month <- main %>%
  mutate(
    entry_month = format(entry_date, "%Y-%m")
  ) %>%
  group_by(entry_month) %>%
  summarise(
    records_entered = n(),
    median_delay = median(reporting_delay, na.rm = TRUE),
    q1 = quantile(reporting_delay, 0.25, na.rm = TRUE),
    q3 = quantile(reporting_delay, 0.75, na.rm = TRUE),
    .groups = "drop"
  )

delay_by_hospital <- main %>%
  group_by(hospital_level) %>%
  summarise(
    N = n(),
    median_delay = median(reporting_delay, na.rm = TRUE),
    q1 = quantile(reporting_delay, 0.25, na.rm = TRUE),
    q3 = quantile(reporting_delay, 0.75, na.rm = TRUE),
    n_gt7 = sum(delay_gt7, na.rm = TRUE),
    pct_gt7 = mean(delay_gt7, na.rm = TRUE) * 100,
    n_gt14 = sum(delay_gt14, na.rm = TRUE),
    pct_gt14 = mean(delay_gt14, na.rm = TRUE) * 100,
    n_gt30 = sum(delay_gt30, na.rm = TRUE),
    pct_gt30 = mean(delay_gt30, na.rm = TRUE) * 100,
    n_gt90 = sum(delay_gt90, na.rm = TRUE),
    pct_gt90 = mean(delay_gt90, na.rm = TRUE) * 100,
    .groups = "drop"
  )

delay_by_visit_month
delay_by_entry_month
delay_by_hospital


# ============================================================
# 21. Sensitivity analyses excluding extreme reporting delays
# ============================================================

p99_delay <- quantile(
  model_dat$reporting_delay,
  0.99,
  na.rm = TRUE
)

dat_excl_p99 <- model_dat %>%
  filter(reporting_delay <= p99_delay)

dat_excl_180 <- model_dat %>%
  filter(reporting_delay <= 180)

fit_delay7_excl_p99 <- glm(
  delay_gt7 ~
    age +
    sex +
    occupation_group +
    hospital_level +
    any_spirometry +
    chest_ct +
    chest_xray +
    aecopd_explicit,
  data = dat_excl_p99,
  family = binomial()
)

fit_delay7_excl_180 <- glm(
  delay_gt7 ~
    age +
    sex +
    occupation_group +
    hospital_level +
    any_spirometry +
    chest_ct +
    chest_xray +
    aecopd_explicit,
  data = dat_excl_180,
  family = binomial()
)

delay_excl_p99_results <- or_table(
  fit_delay7_excl_p99
)

delay_excl_180_results <- or_table(
  fit_delay7_excl_180
)

delay_excl_p99_results
delay_excl_180_results


# ============================================================
# 22. Occupation sensitivity analyses
# ============================================================

# A. Occupation omitted from no-spirometry model
fit_no_spiro_no_occ <- glm(
  absent_spirometry ~
    age +
    sex +
    hospital_level +
    chest_ct +
    chest_xray +
    aecopd_explicit,
  data = model_dat,
  family = binomial()
)

# B. Occupation omitted from delay >7-day model
fit_delay7_no_occ <- glm(
  delay_gt7 ~
    age +
    sex +
    hospital_level +
    any_spirometry +
    chest_ct +
    chest_xray +
    aecopd_explicit,
  data = model_dat,
  family = binomial()
)

# C. Exclude records with missing/uncategorized occupation
occ_complete <- model_dat %>%
  filter(
    occupation_group != "Other/uncategorized"
  ) %>%
  droplevels()

fit_no_spiro_occ_complete <- glm(
  absent_spirometry ~
    age +
    sex +
    occupation_group +
    hospital_level +
    chest_ct +
    chest_xray +
    aecopd_explicit,
  data = occ_complete,
  family = binomial()
)

or_table(fit_no_spiro_no_occ)
or_table(fit_delay7_no_occ)
or_table(fit_no_spiro_occ_complete)


# ============================================================
# 23. Descriptive analysis restricted to documented
#     post-bronchodilator FEV1/FVC <0.70
# ============================================================

post_bd_obstructed <- model_dat %>%
  filter(
    !is.na(post_bd_fev1_fvc),
    post_bd_fev1_fvc < 70
  )

post_bd_obstructed %>%
  summarise(
    n = n(),
    aecopd_n = sum(aecopd_explicit, na.rm = TRUE),
    aecopd_pct = mean(aecopd_explicit, na.rm = TRUE) * 100,
    delay_gt7_n = sum(delay_gt7, na.rm = TRUE),
    delay_gt7_pct = mean(delay_gt7, na.rm = TRUE) * 100,
    median_delay = median(reporting_delay, na.rm = TRUE),
    q1_delay = quantile(reporting_delay, 0.25, na.rm = TRUE),
    q3_delay = quantile(reporting_delay, 0.75, na.rm = TRUE)
  )


# ============================================================
# 24. Marginal age probabilities from spline model
#     for absent spirometry documentation
# ============================================================

age_grid <- data.frame(
  age = seq(
    floor(min(model_dat$age, na.rm = TRUE)),
    ceiling(max(model_dat$age, na.rm = TRUE)),
    by = 1
  )
)

age_predictions <- avg_predictions(
  fit_no_spiro_spline,
  newdata = datagrid(
    model = fit_no_spiro_spline,
    age = age_grid$age,
    grid_type = "counterfactual"
  ),
  by = "age",
  type = "response"

age_predictions


# ============================================================
# 25. Session information
# ============================================================

sessionInfo()
```
